# Biochemical and gene expression analyses character volatile compounds, fatty acids, and physiochemical properties of *Stauntonia obovatifoliola* seed oil^☆^

**DOI:** 10.1016/j.fochx.2025.103221

**Published:** 2025-10-30

**Authors:** Xiaolin Li, Jiqing Zhong, Junhui Zhou, Yanan Wang, Hui Huang

**Affiliations:** a State Key Laboratory for Quality Ensurance and Sustainable Use of Dao-di Herbs, National Resource Center for Chinese Materia Medica, China Academy of Chinese Medical Sciences, Beijing 100700, China; b Key Laboratory of Research and Utilization of Ethnomedicinal Plant Resources of Hunan Province, College of Biological and Food Engineering, Huaihua University, Huaihua 418000, China

**Keywords:** Stauntonia obovatifoliola, Seed oil, Physicochemical characteristics, Volatile compounds, Fatty acid, Carotenoids

## Abstract

*Stauntonia obovatifoliola* subsp. *urophylla* is a promising oilseed crop. This study first systematically evaluated its seed oil including physicochemical properties, fatty acid profile, and volatile compounds, and genes related to carotenoid and fatty acid biosynthesis. The oil had high carotenoid content (77.49 ± 1.11 mg/100 g), moderate iodine value (82.91 ± 2.86 g/100 g), low acid and peroxide values, and antioxidant/antimicrobial activities. Mature seeds contained unsaturated fatty acids (UFAs, 66.68 %), mainly oleic acid (30.93 %) and linoleic acid (21.00 %). A distinctive C19:1N9T component was also detected. Twenty-eight volatile compounds including eight terpenes, eight alkanes, five alcohols, five arenes and two aldehydes. Notably, during rapid oil accumulation phase (July to August), fatty acid biosynthesis genes were elevated, whereas carotenoid synthesis genes were suppressed, suggesting substrate competition. These findings offer help in purposefully exploitation and utilization of *S. obovatifoliola* seeds oil in food, cosmetics and pharmaceuticals.

## Highlights

• *S. obovatifoliola* is a herb and an emerging fruit, as well as a oil-producing plant.
• This is the first investigation into seed oil of *S. obovatifoliola*.
• *S. obovatifoliola* seed oil had high level of carotenoids and unsaturated fatty acids.
• HS-SPME-GC–MS analysis detected 28 volatiles in *S. obovatifoliola* seed oil.
• There may be a substrate competition between carotenoid synthesis and lipid synthesis.

## 1 Introduction

Global demand for edible oils is rising significantly, driven by population growth and shifting dietary preferences (Meijaard et al., 2022). By 2025, global edible oil production must increase by 14 % compared to 2021 levels, reaching 288 million metric tons, to meet the needs of a projected population of 9.7 billion people (IUCN). Moreover, oilseed crops already occupy approximately 35 % of the world's arable land (Food and Agriculture Organization of the United Nations, 2023), making further expansion unsustainable. Therefore, exploring novel, non-conventional oil sources from underutilized seeds, particularly those derived from agricultural by-products, has become imperative. The approach presents a cost-effective and eco-friendly strategy to alleviate the escalating pressure on global edible oil production.

Characteristic the physicochemical and nutritional properties of diverse non-conventional oils is essential for their utilization. Numerous studies have highlighted the high quality and promising application prospects of these oils. For instance, *Paeonia ostii* seed oil is notable for its high α-linolenic acid (ALA) (∼ 45 %) that is associated with various health benefits (Yuan et al., 2022). Grape seed oil is rich in unsaturated fatty acids (UFAs), primarily linoleic acid (53.6–69.6 %) and oleic acid (16.2–31.2 %) (Sabir et al., 2012). Cactus seed oil contains high levels of tocopherols (500–680 mg/kg) and phytosterols (8000–11,100 mg/kg), dominated by γ-tocopherols and β-sitosterol (Nounah et al., 2024). Additionally, tea (*Camellia sinensis*) seed oil is abundant in phenolic compounds, exhibiting strong antioxidant capacity and biological activities such as anti-obesity, anti-inflammation, and antithrombotic effects (Liu et al., 2022). Volatile organic compounds (VOCs) are critical in shaping the sensory attributes of oils. Sun et al. (2024) identified 37 VOCs in blue honeysuckle seed oil via HS-SPME-GC–MS, highlighting aldehydes as the key aroma contributors. Similarly, desirable flavor compounds such as ethyl acetate, 1-pentanol, 2-pentylfuran, and 2-heptanone were detected in green plum seed kernel oil (Xu et al., 2023). The exploitation of such non-conventional oil crops not only enriches oil diversity but also holds significant socio-economic potential, supporting farmers and fostering sustainable rural development.

*Stauntonia obovatifoliola* Hayata subsp. *urophylla* (Hand.-Mazz) (Thereafter referred to as *S. obovatifoliola*) belongs to the *Stauntonia* genus (Lardizabalaceae) and is a perennial woody liana (Zou et al., 2020). The species is widely distributed across multiple provinces of China, including Hunan, Hubei, Guangdong, Guangxi, Jiangxi, Fujian, Zhejiang, etc. (Wu et al., 2013). As a traditional Chinese medicinal plant, its stems and fruits contain abundant bioactive compounds, such as phenolics, triterpenes, sterides and polysaccharides, which are utilized in the treatment of diverse health conditions (Lu et al., 2014; Lu et al., 2015; Peng et al., 2013). *S. obovatifoliola* is widely cultivated in China as a functional fruit, valued for its nutritional composition including sugars, proteins, vitamins, amino acids, mineral elements, and dietary fiber (Li et al., 2022; Wang et al., 2015). In recent years, *S. obovatifoliola* has been recognized as a potential source of edible oil and non-edible products, such as soap, driving its expanded cultivation for economic purposes (Wei et al., 2008). Each fruit contains over 100 seeds*,* with an average fruit weight of 15.4 g (Wei et al., 2008), making the seeds (a major agricultural by-product) a promising candidate for oil extraction. This potential is further supported by the use of seed oil from *Akebia trifoliata* subsp. *australis*, a related species in the same family, which is rich in quality edible oil by local people and dietary supplement in China due to its high UFAs (Jiang et al., 2021; Zhao et al., 2014). Our research team has previously investigated the fatty acids profile and its transcriptional regulation in *Akebia trifoliata* subsp. *australis* seed (Huang et al., 2021). Although *S. obovatifoliola* seed oil has been used as edible oil in certain regions of China, there remains a scarcity of systematic studies on its physiochemical properties, volatile compounds, and fatty acid composition. Both fatty acid profiles and key fat-soluble nutrients (e.g., carotenoids) contribute collectively to the superior quality of oils and fats, and their biosynthesis pathways may be co-regulated. Thus, the genetic basis underlying the biosynthesis of fatty acids and nutritional components in *S. obovatifoliola* remains largely unknown, which significantly hampers efforts in genetic breeding and resource utilization of this species.

This study presents a systematic analysis of the physicochemical characteristics, volatile compounds, fatty acids profile, and biological activities (antioxidant and antimicrobial) of *S. obovatifoliola* seed oil. Moreover, the gene expression patterns of key genes involved in fatty acids and carotenoids biosynthesis across seed developmental stages was analyzed. The results will provide a theoretical and scientific basis for the comprehensive development and utilization of *S. obovatifoliola* seeds oil, and contribute to the agricultural improvement of this underutilized species*.*

## 2 Materials and methods

### 2.1 Plant materials and chemical reagents

The *S. obovatifoliola* used in the study were grown a plantation located at Huitong, Hunan Province, China. Seed development stages were monitored weekly from anthesis to full maturity during the growing season (June–October 2022). Seeds were harvested at five developmental timepoints: 20, 50, 80, 110 and 140 days after anthesis (DAA), corresponding to June 15, July 16, August 15, September 16 and October 15, respectively. These developmental stage samples were designated as JUN (20 DAA), JUL (50 DAA), AUG (80 DAA), SEP (110 DAA) and OCT (140 DAA). For each time point, six fruits from two different trees at the same developmental stage were sampled for seed collection. All samples were immediately frozen in liquid nitrogen and stored at −80 °C until analysis. Seeds harvested at full maturity (OCT, 140 DAA) were specifically used for oil extraction and physicochemical characterization.

Analytical standards for α-carotene, β-carotene, capsanthin, xanthophyll, gallic acid, methyl salicylate and ethyl decanoate were purchased from Sigma-Aldrich (St.Louis, MO, USA). Dichloromethane, n-hexane, cyclohexane, chloroform, acetic acid, sodium thiosulfate standard solution, Folin-Ciocalteu reagent, and 1,1-diphenyl-2-picrylhydrazyl (DPPH) were purchased from Aladdin Biochemical Technology Co. Ltd. (Shanghai, China). All other chemicals used were of analytical grade and obtained from regional suppliers. Total RNA was extracted using the RNAprep Pure Plant Plus Kit (Tiangen, DP441). cDNA was synthesized from 1 μg RNA using the FastKing RT Kit (With gDNase) (Tiangen, KR116). And quantitative PCR was performed with SuperReal PreMix Plus (SYBR Green) (Tiangen, FP205).

### 2.2 Dichloromethane and n-hexane extraction

Oil extraction was referred from the previous literature (Belhoussaine et al., 2024) with minor modification. Specifically, 100 g of seed powder was homogenized with 750 mL dichloromethane or n-hexane and subjected to continuous stirring (300 rpm) for 5 h at room temperature in the dark. The mixture was then filtered, and the solvent was evaporated under reduced pressure using a rotary vacuum evaporator (SHZ-958, Yuhua Ltd., China). The evaporation temperatures were set at 43 °C for dichloromethane and 50 °C for n-hexane, respectively. The obtained oil was stored at 4 °C, and each extraction was replicated three times. The oil yield was calculated according to Eq. (1)

(1) $$\text{oil yield}\;\left(\%\right)=\text{oil weight}/\text{seed weight}\times 100$$

### 2.3 Proteins, iodine value and minerals content determination

The protein content and iodine value (IV) of the *S. obovatifoliola* seed oil were determined according to the methods of GB/T 5009.5-2010 and GB/T 5532-2022, respectively. Minerals content analysis was performed following the method described in Chen et al. (2024). Briefly, 1 mL of oil sample was mixed with 8.0 mL of concentrated HNO_3_ (65 %, *w*/w) using a microwave digesting system for 90 min. The solution was then diluted to a final volume of 10 mL with deionized water. The elements including potassium (K), calcium (Ca), phosphorus (P) and magnesium (Mg) concentrations were quantified by atomic absorption spectrometry (AA 800, PerkinElmer, Germany). All measurements were performed with at least three analytical replicates.

### 2.4 Physicochemical analysis

The acid value (AV) and peroxide value (PV) analyses were conducted immediately after oil extraction. According to GB 5009.229-2016 (National Food Safety Standard of China), AV was determined using the titration method. In this procedure, the oil sample was fully dissolved in a mixture of absolute alcohol:ether = 1:2, *v*/v), and titrated with a standardized potassium hydroxide (KOH) solution. The AV was expressed as the milligrams of KOH required to neutralize 1 g of free fatty acid. Following GB 5009.227-2016 (National Food Safety Standard of China) guidelines, oil sample was fully dissolved in a chloroform:acetic acid (2:3, *v*/v) and then titrated with a standardized sodium thiosulfate solution. The PV was expressed as milliequivalents of active oxygen per kilogram of oil.

### 2.5 Chlorophyll content and browning index (BI)

The chlorophyll content of seed oil was determined following the method of Suri et al. (2022). The oil was dissolved in cyclohexane, and the absorbance at 670 nm was measured using a spectrophotometer. The chlorophyll content was estimated using Eq. (2)

(2) $$\text{Chlorophyll}\;\left(\mathrm{mg}/\mathrm{kg}\right)=\left(A_{670}\times 10^{6}\right)/\left(613\times 100\times \text{density}\right),$$

where A_670_ is the absorbance at 670 nm, and the density was derived from the mass-to-volume ratio of the solution. The BI of seed oil was measured by dissolving in chloroform (1:20 *w*/*v*) as reported by Suri et al. (2022). The absorbance was measured at 420 nm using spectrophotometer to represent the non-enzymatic BI of oils.

### 2.6 Extraction of carotenoids and HPLC profile

Carotenoid extraction and determination were referred to Yang et al. (2023) with slightly modified. For carotenoid extraction, 1 mL n-hexane was added to 1 mL of oil sample, followed by vortexing and thorough mixing. The mixture was then centrifuged at 3000 rpm for 5 min. The n-hexane layer was transferred to a new test tube. This extraction step was repeated twice. Subsequently, the combined n-hexane extract was evaporated in a rotary evaporator (Yamato, Rotary Evaporator, model-RE 801) (<30 °C). Finally, the carotenoid extract was dissolved in 200 μL of methanol and injected to identify the carotenoid profile by HPLC.

The HPLC analysis was performed on a Thermo Scientific™ UltiMate™ 3000 Dual Gradient system (Thermo Fisher Scientific, MA, USA). The carotenoids were separated on a YMC carotenoid S-3 μm column (150 × 4.6 mm) with detection at 450 nm. The mobile phase consisted of methanol (eluent A) and a mixture of methanol: methyl *tert*-butyl ether: water (20,75,5, *v*/*v*/*v*, eluent B). The gradient elution program was as follows: 0 min, 0 % B; 15 min, 61 % B; 25 min, 100 % B; 25.1 min, 0 % B; 30 min, 0 % B at a flow rate of 1 mL/min. The column temperature was maintained at 40 °C. External calibration was used for quantification. Calibration curves for each carotenoid were established under the same chromatographic conditions, all exhibiting good linearity (R^2^ > 0.99) across the concentration ranges studied.

### 2.7 Total phenolic content (TPC) and total flavonoids content (TFC)

The TPC and TFC were measured according to the previously described method (Hao et al., 2023) with slight modifications. The oil extract (0.1 mL), 0.1 mL Folin-Ciocalteu reagent and 1.6 mL distilled water were mixed and incubated for 3 min. Then, 0.2 mL saturated sodium carbonate (Na_2_CO_3_) solution was added. The solution was maintained at room temperature for 2 h in the dark. Absorbance was recorded at 725 nm on ELISA assay system (Infinite M200 pro, Tecan, Switzerland). The TPC content was calculated from a standard curve prepared using gallic acid and expressed as microgram of gallic acid equivalents (GAE) per milliliter of oil extract.

The TFC was quantified using the aluminum nitrate [Al(NO_3_)_3_] spectrophotometric method as described in the TFC assay Kit (Solarbio, BC1330, Beijing, China). Briefly, 200 μL of extract using 80 % methanol (1:20, *w*/*v*) was reacted with 40 μL of 5 % (*w*/*v*) sodium nitrite (NaNO_2_) for 6 min at room temperature, followed by addition of 40 μL of 10 % (w/v) Al (NO_3_)_3_ and incubated for 6 min. The reaction was initiated by adding 400 μL of NaOH (4 %). Following incubation for 15 min, absorbance was measured at 470 nm against a blank. Quantification was performed using a rutin standard curve (0–1.5 mg/mL), with results expressed as rutin equivalents (μg RE/mL of oil extract).

### 2.8 Sample preparation for fatty acids composition assay

The fatty acids composition assay was performed according to the previously described method (Peng et al., 2024) with slight modifications. An appropriate amount of sample was added into a 2 mL centrifuge tube with 1 mL chloroform methanol (2:1, *v*/*v*) solution and 100 mg glass beads, and then was homogenized twice at 60 Hz. After ultrasound extraction for 30 min, the mixture was centrifuged at 12,000 rpm for 5 min at 4 °C. Subsequently, 2 mL 1 % sulfuric acid-methanol solution was added to the supernatant. The esterification was carried out at 80 °C for 30 min, after which 1 mL of n-hexane was added. Then, 5 mL H_2_O was added for washing after vortexing and standing for 5 min. The sample was then centrifuged at 3500 rpm for 10 min at 4 °C. Anhydrous sodium sulfate powder (100 mg) was added supernatant to remove excess water. After vortexing and centrifugation, 300 μL of the 10-fold diluted solution was pipetted into a 2 mL centrifuge tube. Finally, 15 μL methyl salicylate (500 ppm) was added as an internal standard, and the mixture was used for GC–MS analysis.

### 2.9 Assay of fatty acids composition

Gas chromatography (GC) analysis was conducted on a Trace 1300 gas chromatograph (Thermo Fisher Scientific, USA) equipped with a capillary column (Thermo TG-FAME, 50 m × 0.25 mm ID × 0.20 μm). Helium was used as the carrier gas at a constant flow rate of 0.63 mL/min. Sample injection was carried out in split mode (8:1) with an injection volume of 1 μL and an injector temperature of 250 °C. The column oven temperature was programmed as follows: initial temperature of 80 °C held for 1 min, increased to 160 °C at 20 °C/min and held for 1.5 min, then raised to 196 °C at 3 °C/min and maintained for 8.5 min, and finally increased to 250 °C at 20 °C/min and held for 3 min. The temperatures of the ion source and transfer line were set at 300 °C and 280 °C, respectively. Mass spectrometric detection was performed on ISQ 7000 system (Thermo Fisher Scientific, USA) with electron impact ionization mode. Single ion monitoring (SIM) mode was used with the electron energy of 70 eV.

### 2.10 Headspace solid phase microextraction (HS-SPME)-gas chromatography–mass spectrometry (GC–MS) analysis of volatile components

Volatile compounds of *S. obovatifoliola* oil were extracted through HS-SPME, and were analyzed using GC–MS according to the method described by Nie et al. (2024). A total of 1 mL oil sample was transferred into a 20 mL headspace vial sealed with an aluminum crimp cap. The vials were equilibrated at 60 °C water bath for 15 min. The HS-SPME fiber (divinylbenzene/carboxene/polydimethylsiloxane, Supelco Inc., Bellefonte, PA, USA) was exposed to the headspace for 30 min at 60 °C. Subsequently, the fiber was immediately desorbed into the GC injection port at 250 °C for 3 min.

The volatile matter was analyzed by GC–MS (Shimadzu, QP2010, Kyoto, Japan) in splitless injection mode. The initial column temperature was set at 25 °C and held for 3 min, then increased to 80 °C and held for 2 min, followed by heating at 230 °C at 6 °C/min and held for 13 min. Mass spectrometry analysis was operated in electron ionization mode (70 eV) with an ion source temperature of 230 °C and a scan range of 35–500 *m/z*. Volatile compounds were identified based on their retention times and drift time of standards by referring to MS database (NIST2014) with a match threshold ≥80. Ethyl decanoate was used as the internal standard.

The odor activity value (OAV) of each volatile compound was calculated to assess its contribution to the overall flavor. The OAV was calculated with equation (Eq. (3))

(3) OAV=Ci/Ti,

where Ci and Ti represent the relative concentration and odor threshold in the water of the compounds, respectively.

### 2.11 Antioxidant activities

Antioxidant activity was measured based on DPPH radical scavenging ability using the method described by Suri et al. (2022). Oil samples were diluted and prepared in ethanol under ultrasound bath for 15 min to obtain 10, 20, 25, 30, 35 and 50 mg/mL solution. Then, 40 μL of each solution was mixed with 250 μL of 0.2 mM DPPH solution. Then the mixture was vortexed and incubated in dark at room temperature for 30 min. The absorbance was then measured at 517 nm using a spectrophotometer against a blank. The DPPH values were expressed as IC_50_ (mg/mL), defined as the sample concentration required to achieve 50 % radical scavenging activity (RSA). The RSA was measured using the below-mentioned equation (Eq. (4)).

(4) $$\mathrm{RSA}\;\left(\%\right)=\left(1-A_{\text{sample}}/A_{\text{control}}\right)\times 100$$

where A_sample_ represents the absorbance of sample and A_control_ represents the absorbance of control.

### 2.12 Antimicrobial activity

The hexane and dichloromethane extracts of seed oil were subjected to a solvent extraction according to method descried in Section 1.2. Three Gram-negative bacteria [*Escherichia coli*, ATCC25922 (*E. coli*); *Salmonella enterica* subsp. *enterica*, ATCC14028 (*S. enterica*); *Pseudomonas aeruginosa*, ATCC27853 (*P. aeruginosa*)], and one Gram-positive bacteria [*Staphylococcus aureus* subsp. *aureus*, ATCC29213 (*S. aureus*)] were obtained from the China General Microbiological Culture Collection Center, China. *Epidermophyton floccosum*, CBS 566.94 (*E. floccosum*), *Trichophyton rubrum*, ATCC4438 (*T. rubrum*) and *Microsporum gypseum*, CBS118893 (*M. gypseum*) were obtained from Medical Fungal Collection Center of the Chinese Academy of Medical Sciences, China. In addition, *Candida albicans*, ATCC10231 (*C. albicans*) was obtained from Microbiologics, USA. Ceftazidime and penicillin G sodium were used as standards for bacteria, and terbinafine hydrochloride and amphotericin B were used as standards for fungus. The diluted samples were added to a 96-well culture plate, followed by inoculation with bacterial or fungal suspension to a final concentration of 5 × 10^5^ CFU/mL. The solution was incubated for 24 h at 37 °C in antibacterial activity analysis, and incubated for 5 days at 25 °C in antifungal activity analysis. Then, absorbance was measured at 625 nm for calculation of antimicrobial rate (NCCLS, 1999; Favarin et al., 2024). The antimicrobial rate (AR, %) was calculated using the following equation (Eq. (5)).

(5) $$\text{Antimicrobial rate}\;\left(\%\right)=\left(A_{\text{blank}}-A_{\text{sample}}\right)/\left(A_{\text{blank}}\right)\times 100$$

where A_sample_ is the absorbance of the test well and A_blank_ is the absorbance of the medium-only well.

### 2.13 Gene expression analysis and quantitative real-time PCR

RNA sequencing libraries were prepared from purified RNA samples using the Illumina Library Prep Kit (Illumina, San Diego, CA, USA) and sequenced on the Illumina HiSeq X Ten platform. Raw reads were processed to obtain clean reads using Trimmomatic software, followed by *de novo* transcriptome assembly with Trinity. Gene expression levels were quantified in the fragments per kilobase million (FPKM) value using Bowtie 2 (v2.3.1) and RSEM (version 1.3.0). Differentially expressed genes (DEGs) were identified by using DESeq2 (v1.24.0) under the criteria of false discovery rate (FDR) < 0.05 and |fold change (FC)| ≥ 2. Functional annotation of unigenes was performed by BLASTX alignment against NCBI nonredundant protein (Nr) database (https://www.ncbi.nlm.nih.gov/), Swiss-Prot protein database (https://www.uniprot.org/), Kyoto Encyclopedia of Genes and Genomes (KEGG) database (https://www.genome.jp/kegg/), and Cluster of Orthologous Groups of proteins (COG) database (https://www.ncbi.nlm.nih.gov/research/cog/). The specific primers used in qRT-PCR were designed using Primer 3 (Table S1). The PCR amplification was performed with the SYBR Green PCR kit (Tiangen) on the Bio-Rad CFX96 Touch q-PCR System (Bio-rad, CA, USA), following established protocols (Huang et al., 2021). The *SobEF-2* gene was used as the internal reference gene, and relative expression levels were calculated using the 2^-ΔΔCT^ method. All selected genes underwent triplicate analysis to validate RNA-Seq results.

### 2.14 Statistical analysis

Data are presented as means ± standard error. Statistical comparisons were performed with Tukey's test at (95 % confidence level) using *SPSS 22.0* software to determine the levels of significance. Significant differences (*p* < 0.05) among samples were shown by different letters.

## 3 Results and discussion

### 3.1 The oil yield and nutrients content

The extraction yields of *S. obovatifoliola* seed oil were 26.36 ± 2.46 % and 33.78 ± 3.08 % using dichloromethane and n-hexane, respectively. These values are comparable to those reported for *P. ostii* seed (24.12–37.83 %, Yuan et al., 2022) and *Camellia oleifera* seed (21.52–38.86 %, Zeng et al., 2024). The oil yield of *S. obovatifoliola* seed appears lower than or comparable to that of *A. trifoliata* seed (30.21–48.82 %, Su et al., 2021), both members of the Lardizabalaceae family. The protein content of *S. obovatifoliola* seed oil was 1.09 ± 0.20 g/100 g, which was higher than that reported in 28 virgin and six refined olive oils at 7–51 μg/100 g (Hidalgo et al., 2022). Both extraction methods and protein quantification approaches significantly influence the determination results of protein content in vegetable oils (Hidalgo & Zamora, 2006). Minerals are crucial in human health and important indicators of food quality (Juranovic et al., 2003). The nutrition elements determination showed that P (72.82 ± 4.45 mg/kg) was the most abundant mineral in *S. obovatifoliola* seed oil, followed by Ca (47.63.15 ± 0.19 mg/kg), whereas Mg level (25.55 ± 4.36 mg/kg) was lower (Fig. 2B). These mineral elements contribute to the nutrition value of *S. obovatifoliola* seed oil.

### 3.2 Physicochemical analysis

Analysis of physicochemical characteristics of oil is essential for understanding their properties and quality, providing a foundation for the exploration and utilization of woody seed oils. AV and PV are crucial indicators of edible oil quality (Ribeiro et al., 2017). The AV of *S. obovatifoliola* seed oil was 3.71 ± 1.02 mg KOH/g (Table 1), comparable to reported values for *Nigella sativa* (3.84 mg/g, Suri et al., 2022), *Citrus maxima* (3.99 mg/g, Sarkar et al., 2022), and carrot seed oil (3.73 mg/g, Ji et al., 2023). This AV below the 4 mg/g limit set by Chinese national standard for vegetable oils (GB 2716-2018), indicating low free fatty acid content and high oxidative stability. Furthermore, the PV, a key indicator of oxidative rancidity, was 0.11 ± 0.18 meq O_2_/100 g in *S. obovatifoliola* seed oil (Table 1), which is significantly lower than the China national standard limit of 0.25 g/100 g. The result further confirms the oil's low oxidation level and superior oxidative stability. The IV reflects the degree of oil unsaturation, and was 82.91 ± 2.86 g/100 g of *S. obovatifoliola* seed oil, which is comparable to olive oil (74.02–85.60 g/100 g) (Ashokkumar et al., 2018), lower than *P. ostii* seed oil (161.92–204.46 g/100 g) (Liu et al., 2019) and higher than *Anisophyllea boehmii* kernel oil (54.83–58.93 g/100 g) (Nkengurutse et al., 2019). A moderate level of IV of *S. obovatifoliola* seed oil strikes an optimal balance between oxidative stability and health benefits, suggesting broad application potential.

**Table 1:** The physiochemical properties, radical scavenging and antimicrobial activities of *Stauntonia obovatifoliola* seed oil.

Parameters	Value	Parameters	Value
Oil yield (dichloromethane)	26.36 ± 2.46 %	TFC (μg RE/mL)	52.01 ± 4.34
Oil yield (n-hexane)	33.78 ± 3.08 %	Total chlorophylls (mg/kg)	3.04 ± 0.02
Protein content (g/100 g)	1.09 ± 0.20	DPPH (IC_50_, mg/mL)	31.16 ± 0.46
AV (mg KOH/g)	3.71 ± 1.02	*E. coli* (128 μg/mL, AR %)	33.98 ± 0.61
PV (meq O_2_/100 g)	0.11 ± 0.18	*E. floccosum* (128 μg/mL, AR %)	35.50 ± 1.43
IV (g/100 g)	82.91 ± 2.86	*T. rubrum* (128 μg/mL, AR %)	38.22 ± 2.53
BI (Abs_420nm_)	0.75 ± 0.01	*M. gypseum* (128 μg/mL, AR %)	38.92 ± 2.28
TPC (μg GAE/mL)	164.66 ± 11.75		

Note: AV, Acid value; PV, Peroxide value; AR, Antimicrobial rate; BI, Browning index; IV, Iodine value; TPC, Total phenolic content; TFC, Total flavonoids content.

**Fig. 1:**
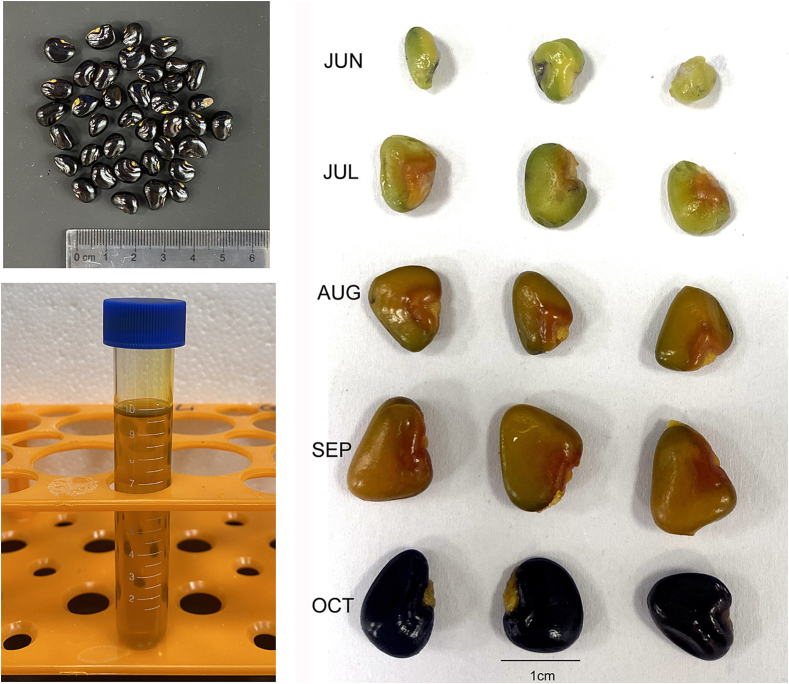
Photos of *S. obovatifoliola* seeds at different developmental stages and seed oil.

**Fig. 2:**
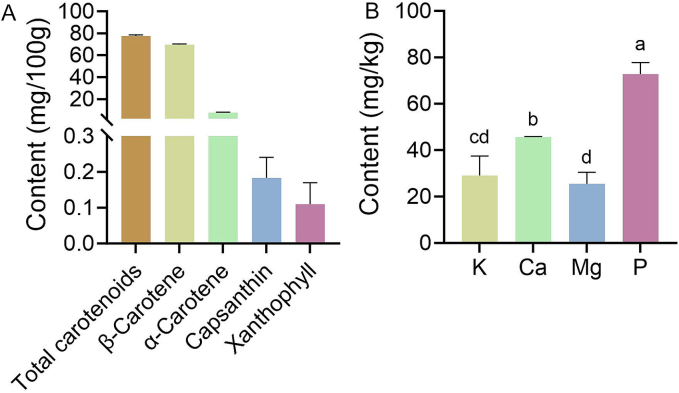
The contents of carotenoids (A) and nutrition elements (B) in *S. obovatifoliola* seed oil.

The *S. obovatifoliola* seed oil exhibited a bright yellowish-amber color with a BI of 0.75 ± 0.01 (Fig. 1). The total carotenoid content was 77.49 ± 1.11 mg/100 g, including β-carotene (69.54 ± 0.79 mg/100 g), α-carotene (7.66 ± 0.33 mg/100 g), capsanthin (0.18 ± 0.07 mg/100 g) and xanthophyll (0.10 ± 0.07 mg/100 g) (Fig. 2). The carotenoids level in *S. obovatifoliola* seed oil was much higher than than reported for many seed oils, such as *Nigella sativa* seed oil (2.48 mg/kg) (Suri et al., 2022) and *Carthamus tinctorius* seed oil (3.9 mg/kg) (Khémiri et al., 2020). The total chlorophyll content was 3.04 ± 0.02 mg/kg (Table 1). Moreover, the oil contained 164.66 ± 11.75 μg GAE/mL of TPC and 52.01 ± 4.34 μg RE/mL of TFC, and exhibited notable antioxidant activity with an IC_50_ value of 31.16 ± 0.46 mg/mL in DPPH assay. Generally, a lower IC_50_ reflects stronger free radical scavenging ability (Yu et al., 2021). The IC_50_ of *S. obovatifoliola* seed oil was significantly lower than that of *Trichosanthes kirilowii* seed shell oil (35.1–44.5 mg/mL) and Chia (*Salvia hispanica* L.) oil (40.3 ± 0.29 mg/mL) (Bodoira et al., 2017), and comparable to *T. kirilowii* seed kernel oil (24.6–34.9 mg/mL) (Xu et al., 2022). Carotenoids and phenolic compounds displayed a highly positive significant (*p* ≤ 0.005) correlation with antioxidant activities of nigella seed oil (Suri et al., 2022). The abundant carotenoid and phenolic compounds might contribute to the higher scavenging ability of free radicals of *S. obovatifoliola* seed oil. Moreover, both carotenoids and phenolic compounds are known to have beneficial effects on human health, including anti-inflammatory and analgesic effects (Zhang et al., 2019).

In addition, antimicrobial activities of *S. obovatifoliola* seed oil extracted with hexane (HE) and dichloromethane (DI) were evaluated. HE showed no antimicrobial effect, whereas DI exhibited different inhibition at 128 μg/mL, with antibacterial activity against *E. coli* (Antimicrobial Rate, AR = 33.98 ± 0.61 %), and antifungal activities against *E. floccosum* (35.50 ± 1.43 %), *T. rubrum* (38.22 ± 2.53 %) and *M. gypseum* (38.92 ± 2.28 %). The result indicated that the seed oils obtained by different extraction methods displayed different antimicrobial activity. The enhanced extraction efficiency of DI over HE results from its medium polarity, which enables better solubilization of antimicrobial phytochemicals (e.g., phenolics and terpenoids), consistent with reports that organic solvents with moderate to high polarity are effective in extracting antibacterial agents than non-polar solvents (Kaczorová et al., 2021). Thus, *S. obovatifoliola* seed oil with antioxidant and antimicrobial activities could be a valuable bioproduct with commercial value and potential applications in skincare and cosmetics industries, similar to the utilization of *Citrus maxima* seed oil in biorefinery and pharmaceutical industries (Sarkar et al., 2022).

### 3.3 Volatile compound analysis

To comprehensively detect volatile compounds in *S. obovatifoliola* seed oil, volatile compounds were determined using HS-SPME-GC/MS. As shown in Table 2, a total of 28 volatile compounds were positively identified, including eight terpenes, eight alkanes, five alcohols, five arene and two aldehydes. Arene (312.16 ± 41.91 μg/kg) represents the most abundant class, followed by terpenes (123.81 ± 18.01 μg/kg), alkanes (98.24 ± 7.54 μg/kg), alcohols (58.22 ± 15.43 μg/kg), and aldehydes (17.51 ± 3.65 μg/kg). The 1,3-dimethylbenzene (182.09 ± 16.00 μg/kg), the most abundant compound in *S. obovatifoliola* seed oil, followed by 1,4-dimethylbenzene (76.02 ± 15.55 μg/kg) and trans-caryophyllene (60.09 ± 4.56 μg/kg). Moreover, to verify the contribution of each volatile compound to the odor profiles in *S. obovatifoliola* seed oil, the odor activity value (OAV) analysis indicated that aldehydes contributed most significantly to the aroma profile, consistent with the fatty acid composition of oil. The increase in aldehydes in seed oil may be attributed to the degradation of UFA (Li et al., 2023). Nonanal exhibited the highest OAV, followed by 1-isopropyl-2-methylbenzene, octane, and dl-limonene. Nonanal (0.72 % ∼ 1.76 %) presented in *S. obovatifoliola* seed oil, and was also the most recognizable smell in grape seed oil (Mildner-Szkudlarz et al., 2021). The dl-limonene is a major contributor to flavor of various berries and their seed oil (Mildner-Szkudlarz et al., 2021; Sun et al., 2024), collectively define the distinctive aroma of *S. obovatifoliola* seed oil.

**Table 2:** The main volatile substances and odor activity value of *S. obovatifoliola* seed oil.

Compounds	RT	Groups	MF	MW	C (μg/kg)	Chemical structures	Odor descriptions	Threshold (mg/kg)	OAV
1,3-Dimethylbenzene	5.27	Arene	C_8_H_10_	106.16	182.09 ± 16.00		fried, medicine, nut, plastic, rancid	1.1	95.82
1,4-Dimethylbenzene	6.00	Arene	C_8_H_10_	106.16	76.02 ± 15.55		cold meat fat, metal, oil, sweet	0.531	79.39
trans-Caryophyllene	25.59	Terpenes	C_15_H_24_	204.35	60.09 ± 4.56		citrus, fried, pepper, spice, wood	0.5	45.07
Octane	3.46	Alkanes	C_8_H_18_	114.23	41.46 ± 2.04		alkane, fat, flower, oil, sweet	0.01	2703.93
Ethylbenzene	5.04	Arene	C_8_H_10_	106.16	43.59 ± 18.21		ethereal, floral, strong, sweet	2.2052	8.20
Dl-limonene	11.42	Terpenes	C_10_H_16_	136.23	25.42 ± 7.35		balsamic, citrus, fragant, fruit, herb	0.01	711.12
Phenylethanol	15.33	Alcohols	C_8_H_10_O	122.16	18.93 ± 4.70		floral, honey, rose	0.75	11.35
Dodecane	18.71	Alkanes	C_12_H_26_	170.33	13.57 ± 2.19		alkane, undesirable	0.01	370.38
cis-Sabinene hydrate	14.62	Alcohols	C_10_H_18_O	154.25	13.10 ± 2.38		balsamic	NA	NA
cis-Sabinene hydrate	13.23	Alcohols	C_10_H_18_O	154.25	15.00 ± 5.51		balsamic	NA	NA
Nonanal	14.99	Aldehydes	C_9_H_18_O	142.24	12.43 ± 2.72		citrus, cucumber, fat, floral, green	0.001	4108.08
Tridecane	22.05	Alkanes	C_13_H_28_	184.36	8.71 ± 1.54		alkane	NA	NA
Undecane	14.79	Alkanes	C_11_H_24_	156.31	10.10 ± 1.98		alkane	2.14	3.36
Germacrene D	27.3	Terpenes	C_15_H_24_	204.35	7.620 ± 1.60		spice, wood	NA	NA
Tetracosane	27.73	Alkanes	C_24_H_50_	338.7	7.51 ± 1.61			NA	NA
α-Caryophyllene	26.54	Terpenes	C_15_H_24_	204.35	6.46 ± 0.45		balsamic, hop, spice, wood	0.16	24.58
1-Heptanol	9.08	Alcohols	C_7_H_16_O	116.2	6.76 ± 1.47		chemical, green, putrid, unpleasant, wood	0.0054	298.19
Nonadecane	32.66	Alkanes	C_19_H_40_	268.5	6.40 ± 2.70		alkane	NA	NA
δ-Cadinene	28.41	Terpenes	C_15_H_24_	204.35	5.95 ± 1.43		medicine, thyme, wood	NA	NA
1-Ethyl-2,3-dimethylbenzene	15.5	Arene	C_10_H_14_	134.22	5.72 ± 2.42			NA	NA
Decane	10.24	Alkanes	C_10_H_22_	142.28	5.21 ± 1.61		alkane	10	5.34
1-Isopropyl-2-methylbenzene	11.26	Arene	C_10_H_14_	134.22	4.74 ± 1.86		citrus, lemon, smoke, wood	0.0005	3294.97
Undecane,3,6-dimethyl-	19.2	Alkanes	C_13_H_28_	184.36	5.26 ± 2.24			NA	NA
1-Octen-3-ol	9.45	Alcohols	C_8_H_16_O	128.21	4.43 ± 1.75		garlic, green leaf, perfume, sweet	0.007	218.45
Squalane	32.79	Terpenes	C_30_H_62_	422.8	6.54 ± 2.61			NA	NA
Phenylacetaldehyde	12.21	Aldehydes	C_8_H_8_O	120.15	5.08 ± 0.93		berry, floral, flower, geranium, honey	0.004	409.94
α-Copaene	24.33	Terpenes	C_15_H_24_	204.35	4.90 ± 1.22		green, spice, wood	NA	NA
β-Elemene	24.81	Terpenes	C_15_H_24_	204.35	4.81 ± 1.72		fresh, fruit, herb, sweet, wax	NA	NA

Note: C, concentration (μg/kg); MF, molecular formula; MW, molecular weight. Odor thresholds in water were obtained from (Nie et al., 2024), (Sun et al., 2024) and (Xiong et al., 2025).

### 3.4 Fatty acids compositions analysis

**Fig. 3:**
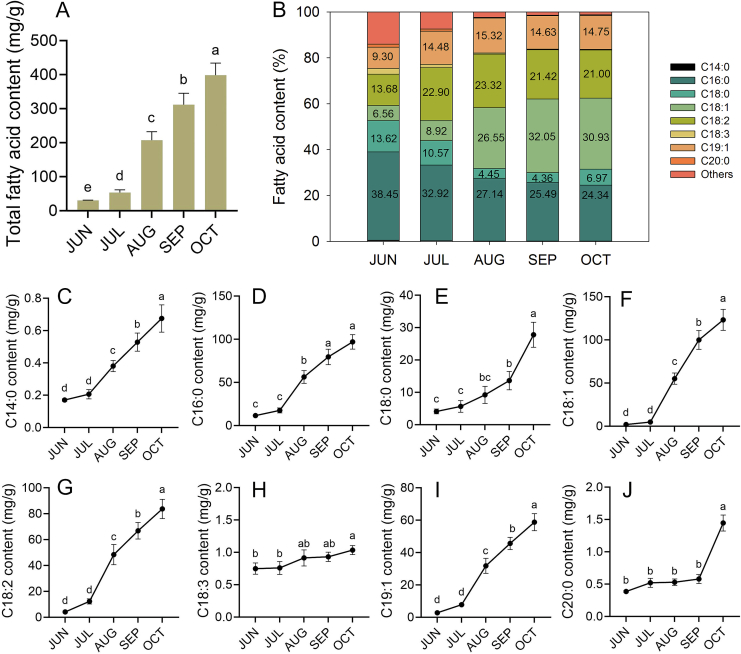
Changes on content and percentage of fatty acids in *S. obovatifoliola* seeds at different developmental stages. (A) Changes in total fatty acid content. (B) Changes in the percentage of fatty acids. (C–J) Changes in the content of key fatty acids.

Fatty acids composition serves as a primary indicator of oil plant quality. During seed development, the color of *S. obovatifoliola* seed shifted from green (20 and 50 DAA) to brown (80 and 110 DAA), and then to black (140 DAA) (Fig. 1). Oil content of *S. obovatifoliola* seed increased significantly from 30.15 ± 0.86 mg/g at 20 DAA (JUN) to 398.86 ± 31.75 mg/g at 140 DAA (OCT), with the most rapid increase between 50 (JUL) and 80 DAA (AUG) (Fig. 3A). Rapid oil accumulation during early seed development stages is commonly observed in numerous woody plant species, such as *A. trifoliata* (Huang et al., 2021), *Prunus pedunculata* (Bao et al., 2021), *P. ostii* (Yuan et al., 2022), and *Xanthoceras sorbifolium* (Zhang et al., 2022). Significant variations in fatty acid content and composition were observed across seed developmental stages, predominantly comprising myristic acid (C14:0), palmitic acid (C16:0), palmitoleic acid (C16:1), heptadecanoic acid (C17:0), stearic acid (C18:0), oleic acid (C18:1N9C), vaccenic acid (C18:1N7), linoleic acid (C18:2N6), ALA (C18:3N3), trans-10-nonadecenoate (C19:1N9T), arachidic acid (C20:0), and carbosaic acid (C20:1) (Table 3). The content of most fatty acids increased significantly during seed development, particularly between JUL and AUG, except for ALA and carbosaic acid. The content of ALA showed a steady and consistent increase with seed development (Fig. 3C–J). Notably, palmitic acid and oleic acid contents changed significantly between JUN and JUL (*p* < 0.05), stabilized in AUG, and differed again in SEP and OCT (Table 3). Additionally, trans-10-nonadecenoate (C19:1N9T) accounted for 14.75 % of total fatty acid, representing a distinctive yet functionally uncharacterized component of *S. obovatifoliola* seed oil.

**Table 3:** The contents of fatty acid (mg/g) of *S. obovatifoliola* seed at five developmental stages.

	C14:0	C16:0	C16:1	C17:0	C18:0	C18:1N9C	C18:1N7	C18:2N6	C18:3N3	C19:1N9T	C20:0	C20:1
JUN	0.17 ± 0.01^Ed^	11.56 ± 0.18^Ac^	0.13 ± 0.01^Eb^	0.21 ± 0.01E^c^	4.10 ± 0.66^Bc^	1.50 ± 0.29^Dd^	0.11 ± 0.02^Ec^	4.03 ± 0.69^Bd^	0.67 ± 0.08^DE^	2.80 ± 0.53^Cd^	0.39 ± 0.02^Eb^	0.88 ± 0.05^DE^
JUL	0.21 ± 0.03^Dd^	17.62 ± 2.57^Ac^	0.15 ± 0.02^Db^	0.22 ± 0.02^Dc^	5.66 ± 1.61^Cc^	4.19 ± 0.51^Cd^	0.26 ± 0.02^Dc^	12.19 ± 1.77^Bd^	0.67 ± 0.08^D^	7.75 ± 0.56^Cd^	0.52 ± 0.06^Db^	0.88 ± 0.01^D^
AUG	0.38 ± 0.03^Cc^	56.31 ± 6.55^Ab^	0.54 ± 0.16^Ca^	0.27 ± 0.02^Cbc^	9.23 ± 2.36^Cbc^	52.32 ± 5.69^Ac^	2.38 ± 0.48^Cb^	48.28 ± 6.96^Ac^	0.82 ± 0.33^C^	31.78 ± 4.16^Bc^	0.53 ± 0.04^Cb^	0.98 ± 0.19^C^
SEP	0.54 ± 0.23^Db^	79.52 ± 8.01^Ba^	0.54 ± 0.02^Da^	0.32 ± 0.04^Db^	13.61 ± 2.53^Db^	96.34 ± 9.52^Ab^	3.27 ± 0.16^Da^	66.73 ± 5.69^Bb^	0.84 ± 0.06^D^	45.63 ± 3.43^Cb^	0.58 ± 0.06^Db^	0.82 ± 0.05^D^
OCT	0.68 ± 0.08^Ea^	96.94 ± 7.54^Ba^	0.40 ± 0.03^Ea^	0.43 ± 0.03^Ea^	27.78 ± 3.47^Da^	120.55 ± 10.76^Aa^	2.28 ± 0.11^Eb^	83.59 ± 6.69^Ba^	0.91 ± 0.05^E^	58.76 ± 4.70^Ca^	1.45 ± 0.11^Ea^	0.91 ± 0.08^E^

Results are represented as mean ± standard error (*n* = 3). The mean values with different uppercase letters indicate significant difference at *p* < 0.05 among different fatty acids. The mean values with different lowercase letters indicate significant difference at *p* < 0.05 among different developmental stages.

The relative proportions of those fatty acids also changed significantly during seed development. Palmitic acid was predominant in immature seed (JUN-AUG), accounting for 38.45 %, 32.92 % and 27.14 %, respectively. In SEP and OCT, oleic acid was the most abundant fatty acid in mature seeds, accounting for 32.05 % in SEP and 30.93 % in OCT, followed by palmitic acid (25.49 % and 24.34 %) and linoleic acid (21.42 % and 21.00 %) (Fig. 3B). This composition resembles that of olive oil, which is also rich in oleic acid (75 % of total triacylglycerols, TAG) followed by palmitic (13.5 %) and linoleic acids (5.5 %) (Unver et al., 2017). By comparison, mature seed oils of *A. trifoliata* and *P. ostii* contain substantially higher proportions of linoleic and ALA (Huang et al., 2021; Yuan et al., 2022).

The UFA/saturated fatty acids (SFA) ratio increased from 0.77 in JUN to 2.11 in mature seeds (OCT), indicating a marked shift toward unsaturation during maturation. It is notable that total fatty acids content and UFA/SFA ratio increased dramatically between JUL and AUG, identifying this phase as a critical period for oil accumulation and compositional conversion in *S. obovatifoliola* seeds.

### 3.5 Gene expression analysis on fatty acids biosynthesis

**Supplementary Fig. S1:**
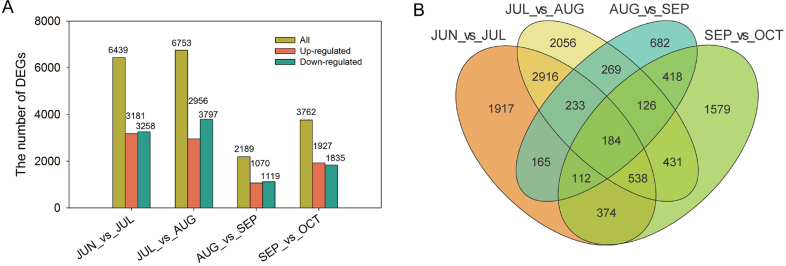
Transcriptome sequencing analysis. (A) The number of differentially expressed genes (DEGs) including up-regulated and down-regulated genes. (B) Venn map of differetially expressed genes by paired comparison.

**Supplementary Fig. S2:**
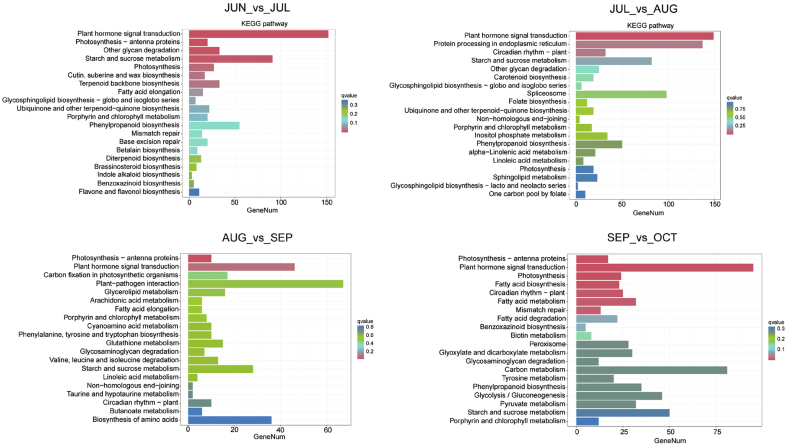
KEGG enrichment analysis on DEGs in paired comparisons. The pathways represented by red bars with *q* ≤ 0.05.

To elucidate the molecular mechanisms underlying fatty acid and carotenoid biosynthesis, we investigated the expression patterns of key genes involved in these pathways across five seed developmental stages. A marked transcriptional shift between JUL (50 DAA) and AUG (80 DAA) stages, evidenced by the highest number of DEGs. This period of pronounced gene expression reprogramming corresponds precisely with the rapid oil deposition (Figs. 1 and S1). Among those identified DEGs, many were enriched in fatty acid metabolic pathways. For instance, ALA and linoleic acid metabolism were found in DEG sets of JUL_vs_AUG and AUG_vs_SEP (Fig. S2).

**Fig. 4:**
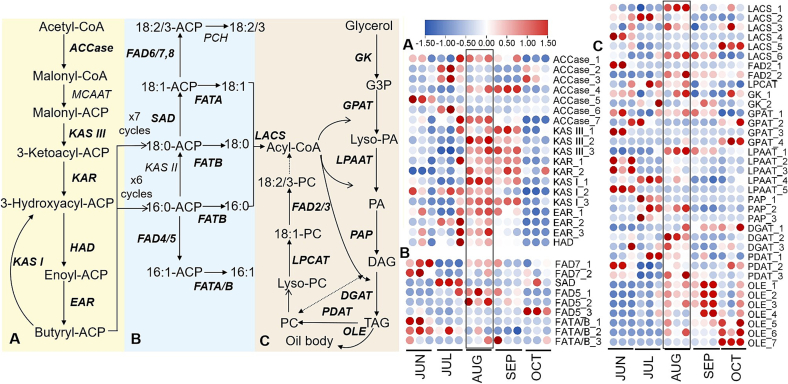
The expression levels of enzymes involved in fatty acids synthesis and TAG assembly in developing *S. obovatifoliola* seeds. (A–C) Catalytic pathways and their corresponding gene expression heatmaps.

**Supplementary Fig. S3:**
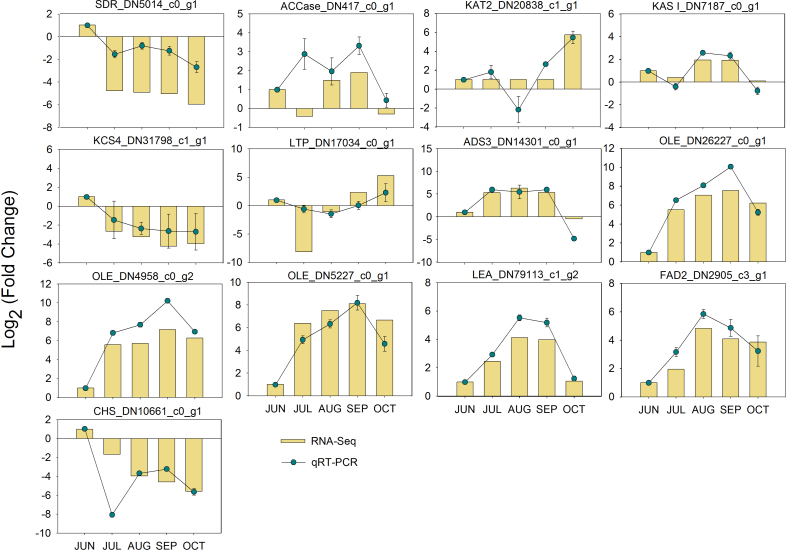
The expression level of 13 selected DEGs in qRT-PCR. Data are average values ± SE (Standard Error) (n = 3) of three biological replicates.

Through detailed analysis of gene expression patterns in the fatty acid biosynthesis pathway, we identified six *acetyl-CoA carboxylases* (*ACCases*) genes were highly expressed in JUL and AUG (Fig. 4A). Overexpressing *ACCase,* a rate-limiting enzyme in fatty acid synthesis, could up-regulate lipid biosynthesis (Chaturvedi et al., 2021). Nearly all DEGs encoding fatty acid synthase (FAS) complex, including *β-ketoacyl-ACP synthase* (*KAS*), *β-ketoacyl-ACP reductase* (*KAR*), *β-hydroxyacyl-ACP dehydratase* (*HAD*), and *enoyl-ACP reductase* (*EAR*), showed maximal transcription in AUG, followed by JUL. The transcription peak of core fatty acid biosynthetic genes coincided with the rapid accumulation of seed oil, a pattern consistent with that reported in *A. trifoliata* seeds (Huang et al., 2021). KAS I catalyzes the elongation of *de novo* fatty acids, and its mutation results in significant reduction in fatty acid contents in seeds (Wu & Xue, 2010). EAR is a key enzyme in *de novo* fatty acid biosynthesis and exhibits the highest expression during the early stages of sunflower seed development (González-Thuillier et al., 2015). We inferred that the significant up-regulation of *ACCase* and *FAS* at AUG and JUL had close relationship with a rapid increase of oil content in *S. obovatifoliola* seeds at AUG. Moreover, *stearoyl-CoA desaturase* (*SAD*), a key *fatty acid desaturase* (*FAD*), exhibited high expression in JUL (Fig. 4B), which may contribute to the rapid accumulation of UFAs in *S. obovatifoliola* seeds by 80 DAA. Similar two *AtrSADs* expression patterns have been linked to high oleic acid content in developing *A. trifoliata* seeds (Huang et al., 2021). The SADs catalyze the first desaturation step, producing the monounsaturated fatty acid (MUFA) oleic acid, which can then be further desaturated to form polyunsaturated fatty acids (PUFAs) (Cai, 2020). The *FAD2_2* expression was highest in AUG followed by SEP and OCT (Fig. 4C). *FAD2* plays a crucial role in PUFA synthesis (Hernández et al., 2019), and the suppression of *FAD2* expression by siRNA leads to low linoleic acid content (5.5 %) in olive oil (Unver et al., 2017). Previous studies indicated that elevated temperatures inhibit FAD2 activity and reduce PUFA accumulation in some species (Martínez-Rivas et al., 2003; Schulte et al., 2013). Many woody species exhibit increased PUFA levels during seed development under rising temperatures, potentially due to sustained high *FAD2* expression (Huang et al., 2021; Wang et al., 2024; Yuan et al., 2022). The differential expression of *FAD2* genes across plant tissues and species may result in distinct fatty acid accumulation profiles. In addition, three *long-chain acyl-CoA synthetases* (*LACSs)*, three *phosphatidic acid phosphatases* (*PAPs*), two *diacylglycerol acyltransferases* (*DGATs*) and a *lysophosphatidylcholine acyltransferase* (*LPCAT*) were also highly expressed in JUL and AUG. *LACS* is involved in facilitating fatty acids transport and its conversion to acyl-CoA (Tranbarger et al., 2011). Haslam et al. (2020) reported that overexpression of endogenous *DGAT* leads to higher lipid yields and elevated levels of docosahexaenoic acid (DHA) in triacylglycerol. It is noteworthy that all seven identified *oleosin* (*OLE*) genes, encoding oil body proteins essential for TAG packaging and oil body formation, showed a substantial increase and significantly higher expression in seeds from AUG to OCT compared to earlier stages (Fig. 4C), as confirmed by qRT-PCR (Fig. S3). Increased expression of fatty acids biosynthesis genes and *oleosins* synergistically drives high oil accumulation in *Jatropha* kernels (∼63 %) (Ha et al., 2019). Collectively, the coordinated high expression of *ACCase* and *FAS* genes, effective fatty acids desaturation by *SAD* and *FAD2*, and high level of *OLEs* enable efficient oil accumulation, especially UFAs, in developing *S. obovatifoliola* seeds.

### 3.6 Gene expression analysis on carotenoids biosynthesis

**Fig. 5:**
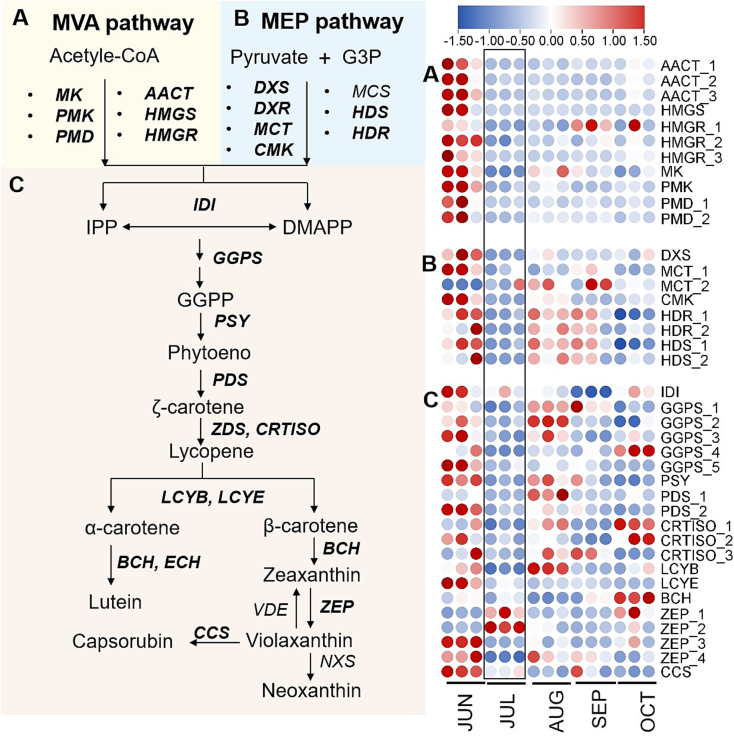
The expression levels of enzymes involved in carotenoids synthesis developing *S. obovatifoliola* seeds. (A–C) Catalytic pathways and their corresponding gene expression heatmaps.

**Fig. 6:**
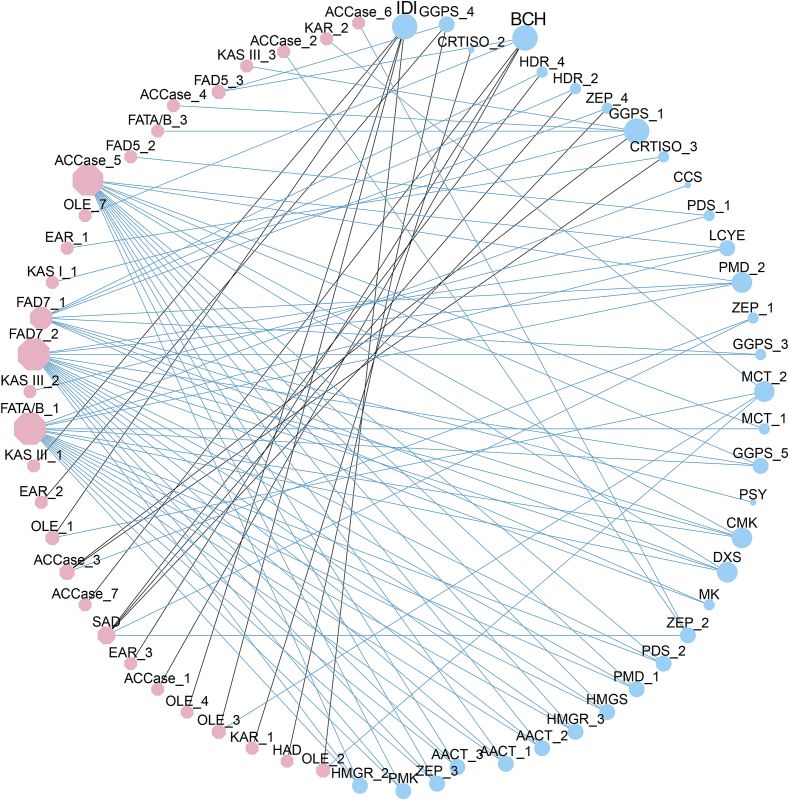
The interaction network between DEGs involved in fatty acid and carotenoid biosynthesis pathways. Pink octagons represent fatty acid biosynthetic genes, blue circles represent carotenoid biosynthetic genes. The size of the graph is positively correlated with its connectivity. Grey lines indicate negative regulation. Blue line represents positive regulation. (For interpretation of the references to color in this figure legend, the reader is referred to the web version of this article.)

Our analysis demonstrated that *S. obovatifoliola* seed oil exhibits remarkably high carotenoids content (77.49 ± 1.11 mg/100 g). To explore its molecular basis, we reconstructed the cartenoids biosynthesis pathway, as depicted in Fig. 5. Carotenoids are derived from C_5_-precursor, produced through the mevalonate (MVA) and plastidial 2-*C*-methyl-D-erythritol 4-phosphate (MEP) pathways (Schüler et al., 2021). All identified DEGs involved in MVA pathway were highly expressed in JUN (Fig. 5A), and decreased thereafter, including *Acetyl-CoA C-Acetyltransferase* (*AACT*), *3-hydroxy-3-methylglutaryl-CoA synthase* (*HMGS*), *3-hydroxy-3-methylglutaryl-CoA reductase* (*HMGR*), *mevalonate kinase* (*MK*), *phosphomevalonate kinase* (*PMK*) and *phosphomevalonate decarboxylase* (*PMD*). In the MEP pathway, nearly all DEGs were highly expressed in JUN (Fig. 5B), and then were down-regulated in JUL, followed by a up-regulation in AUG, including *1-deoxy-D-xylulose 5-phosphate synthase* (*DXS*), *2-C-methyl-D-erythritol 4-phosphate cytidylyltransferase* (*MCT*), *4-diphosphocytidyl-2-C-methyl-D-erythritol kinase* (*CMK*), *hydroxymethylbutenyl diphosphate reductase* (*HDR*) and *hydroxymethylbutenyl diphosphate synthase* (*HDS*). A similar expression pattern were observed for *geranylgeranyl pyrophosphate synthase* (*GGPS*) and *phytoene synthase* (*PYS*) that catalyze the synthesis and condensation of geranylgeranyl pyrophosphate (GGPP) respectively, as well as *phytoene desaturase* (*PDS*) and *carotenoid isomerase* (*CRITS*) that are responsible for a series of sequential desaturation (Fig. 5C). Moreover, *lycopene ε-cyclase* (*LYCE*), *lycopene β-cyclase* (*LYCB*), *β-carotene hydroxylase* (*BCH*), two *zeaxanthin epoxidases* (*ZEPs*) and *carotenoid cleavage dioxygenase* (*CCS*) genes were highly expressed in JUN but suppressed in JUL. Consequently, most carotenoids biosynthesis genes were down-regulated in JUL, while numerous fatty acids biosynthetic genes were up-regulated, coinciding with a marked acceleration of seed oil accumulation. Previous studies in algas have confirmed a competitive relationship between carotenoid and fatty acids synthesis pathways, as both utilize acetyl-CoA and NADPH as common precursors and cofactors (Bi et al., 2023; Schüler et al., 2021; Zhu et al., 2022). Similarly, in *S. obovatifoliola* seeds, a potential metabolic competition appears to occur between carotenoid and lipid biosynthesis pathways during seed development. Carbon flux was directed toward fatty acids synthesis, likely mediated by the downregulation of carotenoid biosynthesis-related genes in July, and the subsequent upregulation of fatty acids synthesis-related genes in August. To further identify key candidate genes regulating the potential metabolic competition, we investigated the correlations between DEGs involved in fatty acid and carotenoid biosynthesis (R^2^ ≥ 0.85, *p* ≤ 0.05) (Fig. 6). Pearson correlation analysis revealed that *IDI* and *BCH* were strongly negatively correlated with several fatty acid biosynthetic genes, including *KAS III_1*, *OLE_1, OLE_2, OLE_3, OLE_4, OLE_7*, *ACCase_1*, *ACCase_7*, *EAR_3* and *FAD5_3*. These result suggest that the negative regulatory relationships among these genes may contribute to the metabolic competition between fatty acid and carotenoid biosynthesis pathways, which is a hypothesis that warrants further validation.

## 4 Conclusions

In this study, physicochemical characteristics, volatile compounds, fatty acids profile, antioxidant and antimicrobial activities of *S. obovatifoliola* seed oil, as well as candidate genes involved in the regulation of fatty acids and carotenoids biosynthesis, were investigated for the first time. Our research revealed that *S. obovatifoliola* seed oil was characterized by high level of carotenoids, particularly β-carotene, low AV and PV, and promising antioxidant and antimicrobial activities. A moderate level of IV (82.91 ± 2.86 g/100 g) of the seed oil strikes an optimal balance between oxidative stability and health benefits, making it suitable for diverse applications. A total of 28 volatile compounds were identified in *S. obovatifoliola* seed oil. Moreover, UFAs accounted for 66.68 % in *S. obovatifoliola* seed oil, mainly including oleic acid and linoleic acid. Notably, gene expression analysis suggested a potential substrate competition between the carotenoid and fatty acids biosynthesis in *S. obovatifoliola* seeds. The results will provide a theoretical and scientific basis for the comprehensive development and utilization of *S. obovatifoliola* seeds oil, and agricultural improvement of this underutilized species.

## CRediT authorship contribution statement

**Xiaolin Li:** Writing – original draft, Resources, Methodology, Funding acquisition, Data curation. **Jiqing Zhong:** Resources, Methodology, Data curation. **Junhui Zhou:** Methodology, Data curation. **Yanan Wang:** Methodology, Data curation. **Hui Huang:** Writing – review & editing, Funding acquisition, Data curation, Conceptualization.

## Declaration of competing interest

The authors declare that they have no known competing financial interests or personal relationships that could have appeared to influence the work reported in this paper.

## Data Availability

Data will be made available on request. The raw data of RNA-seq were uploaded to the NCBI Sequence Read Archive with the accession number PRJNA1119212 (https://www.ncbi.nlm.nih.gov/object/PRJNA1119212).

## Appendix A Supplementary data

Supplementary material 1 Primers used in this study. Supplementary material 1
