# Supplementary material for: Biochemical and gene expression analyses character volatile compounds, fatty acids, and physiochemical properties of Stauntonia obovatifoliola seed oil
Source: Food Chem X. 2025 Oct 30;31:103221. doi: 10.1016/j.fochx.2025.103221 (PMC12630333; doi:10.1016/j.fochx.2025.103221)
Supplement: Supplementary material 1 — Primers used in this study. [file mmc4.docx]

Table S1. Primers used in this study.

| Gene ID | Gene | Forward primer 5’to 3’ | Reverse primer 5’to 3’ |
| --- | --- | --- | --- |
| TRINITY_DN5014_c0_g1 | Short-chain dehydrogenase reductase | GTAGCGAAGGACAGATGG | AAACCTGAGGGTGAGACA |
| TRINITY_DN417_c0_g1 | Acetyl-CoA carboxytransferase | AGAACTCGGCTGGGCTAC | AACGTGCATAAGTGTCAGATAA |
| TRINITY_DN20838_c1_g1 | 3-ketoacyl-CoA thiolase 2 | GGCATCTGTTTGTTTGGC | TCTGGGTGGGTATCCTTG |
| TRINITY_DN7187_c0_g1 | 3-oxoacyl-[acyl-carrier-protein] synthase I | GATTGCCTCCGTTACTGC | AGACCACCCATGCCTGTT |
| TRINITY_DN31798_c1_g1 | 3-ketoacyl-CoA synthase 4 | CTTGCTTGCGAGTTTGTA | TCAGGTAATCTTCGGCTA |
| TRINITY_DN17034_c0_g1 | Non-specific lipid-transfer protein | AAAACGGTTCAGACAGAC | GAGTGGAGATAACGGATG |
| TRINITY_DN14301_c0_g1 | Palmitoyl-monogalactosyldiacylglycerol delta-7 desaturase | CCCCAGTTGAAGGTGAAA | GTGGAGGTGAAGAACAAGAAGA |
| TRINITY_DN26227_c0_g1 | Oleosin | ATCTTACTCACTCTTGCTGGTC | AGCCCGATTATGATGGAA |
| TRINITY_DN4958_c0_g2 | Oleosin | CCCCATCGACATCACAAA | TCCGACCTTCACCACCTC |
| TRINITY_DN5227_c0_g1 | Oleosin | GGTGCTTATGTTGACTGG | TCTGCTCTAGGTGGACGT |
| TRINITY_DN79113_c1_g2 | Late embryogenesis abundant protein | TTACCCGACCAAAGACGC | AAATGGCAGCACCGACAA |
| TRINITY_DN2905_c3_g1 | delta(12)-fatty-acid desaturase | CAACAGTGGATAGGGATTA | GTTTGATTGCCTTAGTCG |
| TRINITY_DN17034_c0_g1 | Non-specific lipid-transfer protein | AAAACGGTTCAGACAGAC | GAGTGGAGATAACGGATG |
| TRINITY_DN10661_c0_g1 | Chalcone synthase | GTTCCAATCGCCATAACC | GCATCAAAGCATCCACCT |
| TRINITY_DN1212_c1_g1 | Translation elongation factor | CGCAAACACCCTCAACGC | GCTTCACGGGAGAACGAC |
